# Supplementary material for: Very‐low‐carbohydrate diet enhances human T‐cell immunity through immunometabolic reprogramming
Source: EMBO Mol Med. 2021 Jun 21;13(8):e14323. doi: 10.15252/emmm.202114323 (PMC8350890; doi:10.15252/emmm.202114323)
Supplement: Supplementary file 1 — Appendix [file EMMM-13-e14323-s005.pdf]

Appendix

Very Low Carbohydrate Diet Enhances Human T Cell Immunity through Immunometabolic Reprogramming

Simon Hirschberger[1,2,†], Gabriele Strauß[1,2,†], David Effinger[1,2], Xaver Marstaller[1], Alicia Ferstl[1], Martin Bernhard Müller[1,2], Tingting Wu[1], Max Hübner[1,2], Tim Rahmel[3], Hannah Mascolo[1], Nicole Exner[4], Julia Heß[5,6], Friedrich Wilhelm Kreth[1], Kristian Unger[5,6], Simone Kreth[1,2,*]

† These authors contributed equally.

1 Walter Brendel Center of Experimental Medicine, Ludwig-Maximilian-University München (LMU), Munich, 81377, Germany.

2 Department of Anaesthesiology and Intensive Care Medicine, Research Unit Molecular Medicine, LMU University Hospital, Ludwig-Maximilian-University München (LMU), Munich, 81377, Germany.

3 Department of Anesthesia, Intensive Care Medicine and Pain Therapy, University Hospital Knappschaftskrankenhaus Bochum, Bochum, 44892, Germany.

4 Chair of Metabolic Biochemistry, Biomedical Center (BMC), Faculty of Medicine, Ludwig-Maximilian-University München (LMU), Munich, 81377, Germany.

5 Helmholtz Center Munich, Research Unit Radiation Cytogenetics, Neuherberg, 85764, Germany.

6 Department of Radiation Oncology, LMU University Hospital, Ludwig-Maximilian-University München (LMU), Munich, 81377, Germany.

***** Correspondence: Simone Kreth, MD, PhD. Walter Brendel Center of Experimental Medicine, Ludwig-Maximilian-University München (LMU). Marchioninistreet 68, 81377, Munich, Germany. simone.kreth@med.uni-muenchen.de. +49 - 89 - 2180 76508

Appendix

Appendix Figure S1 No T cell immunostimulatory effect of BHB.

Appendix Figure S2 No nonspecific immune stimulation through ketogenic diet *in vivo*.

Appendix Table 1: qRT-PCR Primer and Probes

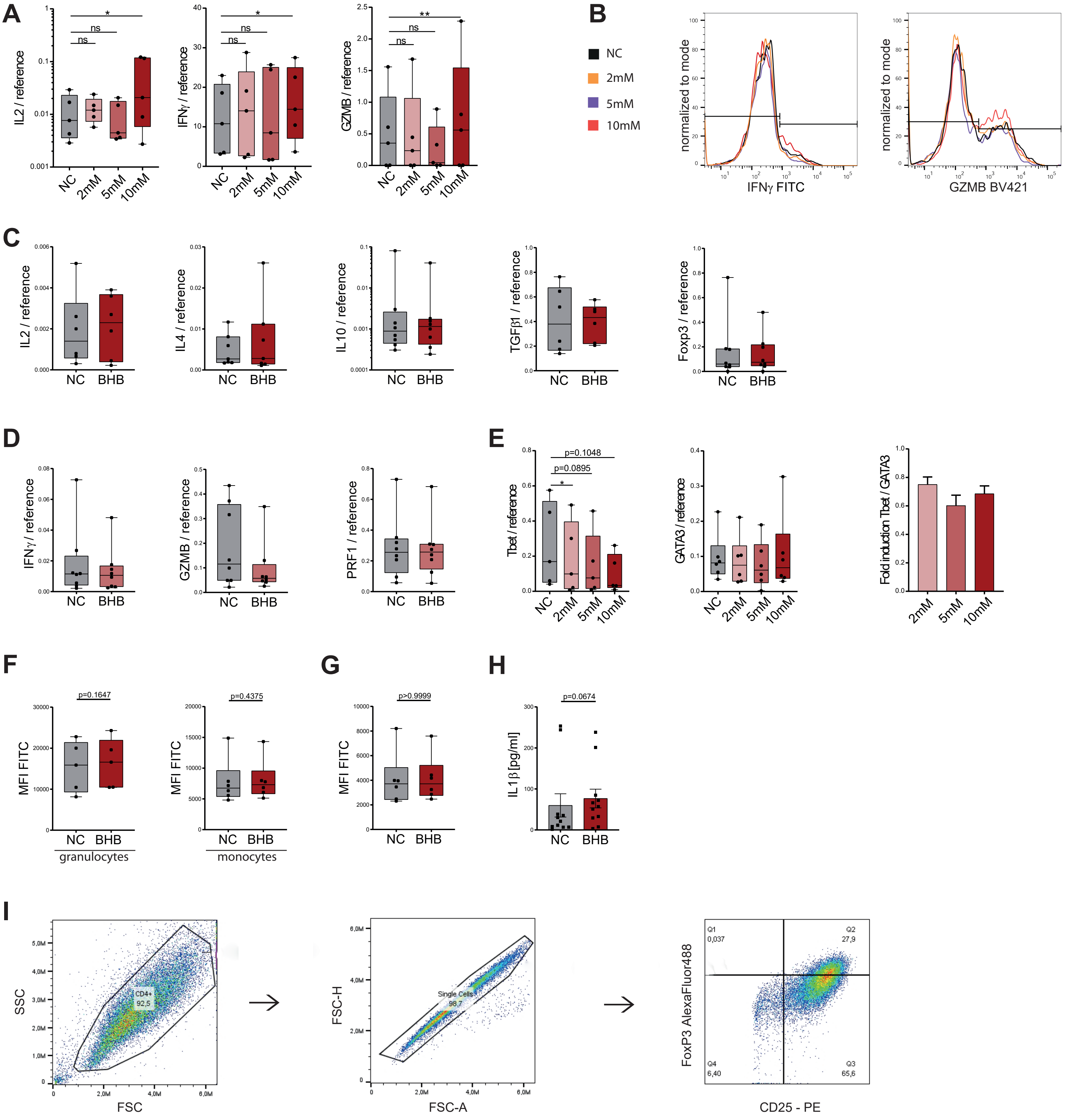
A
IL2 / reference
IFNγ / reference
GZMB / reference
ns
*
**
NC
2mM
5mM
10mM
B
NC
2mM
5mM
10mM
normalized to mode
IFNγ FITC
GZMB BV421
C
IL2 / reference
IL4 / reference
IL10 / reference
TGFβ1 / reference
Foxp3 / reference
NC
BHB
D
IFNγ / reference
GZMB / reference
PRF1 / reference
E
Tbet / reference
GATA3 / reference
Fold induction Tbet / GATA3
p=0.1048
p=0.0895
F
MFI FITC
p=0.1647
p=0.4375
granulocytes
monocytes
G
p>0.9999
H
IL1β [pg/ml]
p=0.0674
I
SSC
FSC
CD4+
92,5
FSC-H
FSC-A
Single Cells
98,7
FoxP3 AlexaFluor488
CD25 - PE
Q1
0,037
Q2
27,9
Q3
65,6
Q4
6,40

Appendix Figure S1 No T cell immunostimulatory effect of BHB.

Human peripheral blood mononuclear cells (PBMCs) were cultivated for 48h in RPMI containing 80mg/dl glucose (NC) and supplemented with 2mM, 5mM and 10mM (Figure S1a/b/e) or only 10mM beta-hydroxybutyrate (BHB | all others). Human whole blood cells were incubated at 37°C for three hours and supplemented with 10mM BHB. **A** Expression of IL2, IFNγ and GZMB mRNA relative to endogenous controls. T cell stimulation was performed through CD3/CD28 Dynabeads at a bead:cell ratio of 1:8. Human pan T cell RNA was isolated after 48 hours, n=5 biological replicates. **B** Flow cytometric quantification of intracellular IFNγ and GZMB protein expression in human Pan T cells cultivated with varying concentrations of BHB as indicated. T cell stimulation as described above. Histogram plot, representative of three individual experiments from three biological replicates. **C** mRNA expression of $CD4^+$ cytokines IL2, IL4, IL10, TGFβ1 and Foxp3 in native human T cells relative to internal controls, n=6/7/9/6/8 biological replicates. **D** mRNA expression of $CD8^+$ cytokines IFNγ, GZMB and PRF1 in native human T cells relative to internal controls. n=9/8/8 biological replicates. **E** Th_1/Th_2 cell transcription factors Tbet and GATA3 quantified via RT-qPCR in human Pan T cells cultivated with varying concentrations of BHB as indicated and the respective ratio of fold induction Tbet/GATA3 mRNA expression, n=6 biological replicates. **F** Phagocytic activity in human granulocytes and monocytes incubated with green E. coli, indicated by mean fluorescence intensity (MFI) FITC, n=6 biological replicates. **G** Quantification of cellular Respiratory Burst after LPS stimulation, using CellROX dye, indicated by mean fluorescence intensity (MFI) FITC in human granulocytes, n=6 biological replicates. **H** IL1β protein expression in the supernatant of stimulated PBMCs, n=13 biological replicates. **I** Representative gating strategy for flow cytometric quantification of human T_{reg} after 5 days of T_{reg} differentiation. Data depicted as mean ± SEM (protein data) and box plots with median, twenty-fifth and seventy-fifth percentiles and range (all other). Dots indicating individual values. *$p<0.05$, **$p<0.01$, ***$p<0.001$, paired t-test/ Wilcoxon matched-pairs signed rank test, as appropriate.

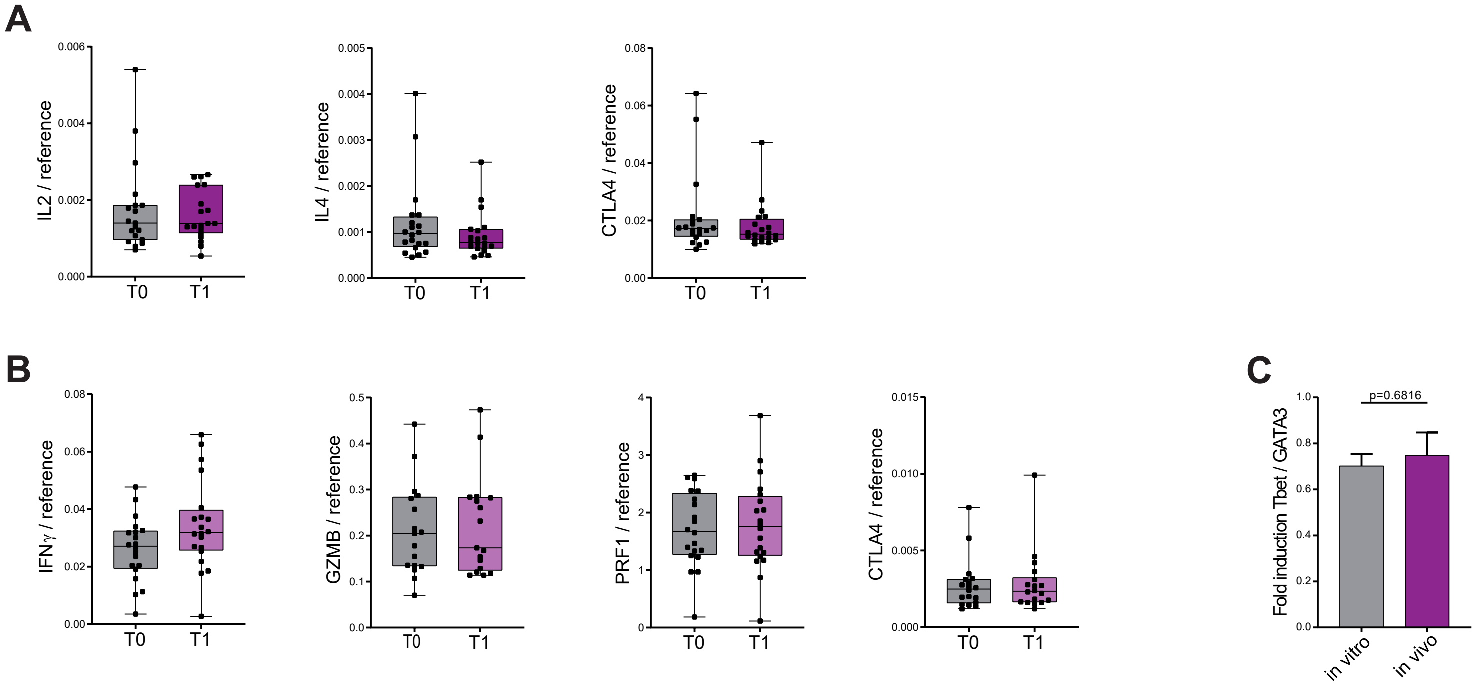
A
IL2 / reference
0.000
0.002
0.004
0.006
T0
T1
IL4 / reference
0.000
0.001
0.002
0.003
0.004
0.005
T0
T1
CTLA4 / reference
0.00
0.02
0.04
0.06
0.08
T0
T1
B
IFNγ / reference
0.00
0.02
0.04
0.06
0.08
T0
T1
GZMB / reference
0.0
0.1
0.2
0.3
0.4
0.5
T0
T1
PRF1 / reference
0
1
2
3
4
T0
T1
CTLA4 / reference
0.000
0.005
0.010
0.015
T0
T1
C
Fold induction Tbet / GATA3
0.0
0.2
0.4
0.6
0.8
1.0
p=0.6816
in vitro
in vivo

Appendix Figure S2 No nonspecific immune stimulation through ketogenic diet *in vivo*.

44 healthy volunteers conducted a three-week ketogenic diet (KD) with a limited carbohydrate consumption of <30g per day. Blood was taken and analyzed before starting the diet (T_0) and again after three weeks of strict adherence to the diet (T_1). PBMCs were isolated. $CD4^+$/$CD8^+$ T cells were separated via magnetic cell labeling. **A** IL2, IL4 and CTLA4 mRNA expression in unstimulated $CD4^+$ T cells, n=20 individual human subjects. Quantification cycles for IL10 have been above the detection threshold. **B** IFNγ, GZMB, PRF1 and CTLA4 mRNA expression in unstimulated $CD8^+$ T cells, n=20 individual human subjects. **C** Ratio of fold induction Tbet/GATA3 mRNA expression in vitro (BHB/NC) and in vivo (T1/T0), n=6. Data depicted as mean with standard error mean (S2c) or box plots with median, twenty-fifth and seventy-fifth percentiles and range (all other). Dots indicating individual values. Unpaired t-test (Figure S2c), Paired t-test/ Wilcoxon matched-pairs signed rank test, as appropriate.

Appendix Table S1: qRT-PCR Primer and Probes

Target	Primer for	Primer rev	Probe
TBP	5’ GAACATCATGGATCAGAACAACA 3’	5’ ATAGGGATTCCGGGAGTCAT 3’	87
SDHA	5’ GAGGCAGGGTTTAATACAGCA 3’	5’ CCAGTTGTCCTCCTCCATGT 3’	132
b-Aktin	5’ CCAACCGCGAGAAGATGA 3’	5’ CCAGAGGCGTACAGGGATA 3’	64
TGFB1	5’ ACTACTACGCCAAGGAGGTCA 3’	5’ TGCTTGAACTTGTCATAGATTTCG 3’	31
PRF1	5’ CACTCACAGGCAGCCAACT 3’	5’ GGGAGTGTGTACCACATGGA 3’	26
GZMB	5’ GGGGGACCCAGAGATTAAAA 3’	5’ CCATTGTTTCGTCCATAGGAG 3’	37
IL2	5’ AAGTTTTACATGCCCAAGAAG 3’	5’ AAGTGAAAGTTTTTGCTTTCAGCT 3’	65
IL4	5’ TGCCTCACATTGTCACTGC 3’	5’ GCACATGCTAGCAGGAAGAAC 3’	38
IL6	5’ GATGAGTACAAAAGTCCTGATCCA 3’	5’ CTGCAGCCACTGGTTCTGT 3’	7
IL7	5’ TATGGGCGGTGAGAGCTT 3’	5’ AGGGGAGGAAGTCCAAAGATA 3’	15
IL7R	5’ GCTTTTGAGGACCCAGATGT 3’	5’ AGGCAGTTTACCTCCACGAG 3’	9
IL10	5’ TGCCTTCAGCAGAGTGAAGA 3’	5’ GCAACCCAGGTAACCCTTAAA 3’	67
IL15	5’ GGAGTGAAGCTACAGACCTTCG 3’	5’ TTGAAGAGCTGGCTATGG 3’	66
IFNy	5’ GGCATTTTGAAGAATTGGAAAG 3’	5’ TTTGGATGCTCTGGTCATCTT 3’	21
Foxp3	5’ CCTTGCCCCACTTACAGG 3’	5’ CCACCGTTGAGAGCTCCT 3’	65
CTLA4	5’ TCACAGCTGTTTCTTTGAGCA 3’	5’ AGGCTGAAATTGCTTTTCACA 3’	21
Tbet	5’ TGGGTGCAGTGTGGAAAG 3’	5’ TCCTGTGTTGGGGGAGTC 3’	72
GATA3	5’ CTCATTAAGCCCAAGCGAAG 3’	5’ TCTGACAGTTCGCACAGGAC 3’	71
IL8	Roche RealTime Ready Single Assay | ID 103136		
