# Supplementary material for: Very‐low‐carbohydrate diet enhances human T‐cell immunity through immunometabolic reprogramming
Source: EMBO Mol Med. 2021 Jun 21;13(8):e14323. doi: 10.15252/emmm.202114323 (PMC8350890; doi:10.15252/emmm.202114323)
Supplement: Supplementary file 2 — Expanded View Figures PDF [file EMMM-13-e14323-s001.pdf]

Expanded View Figures

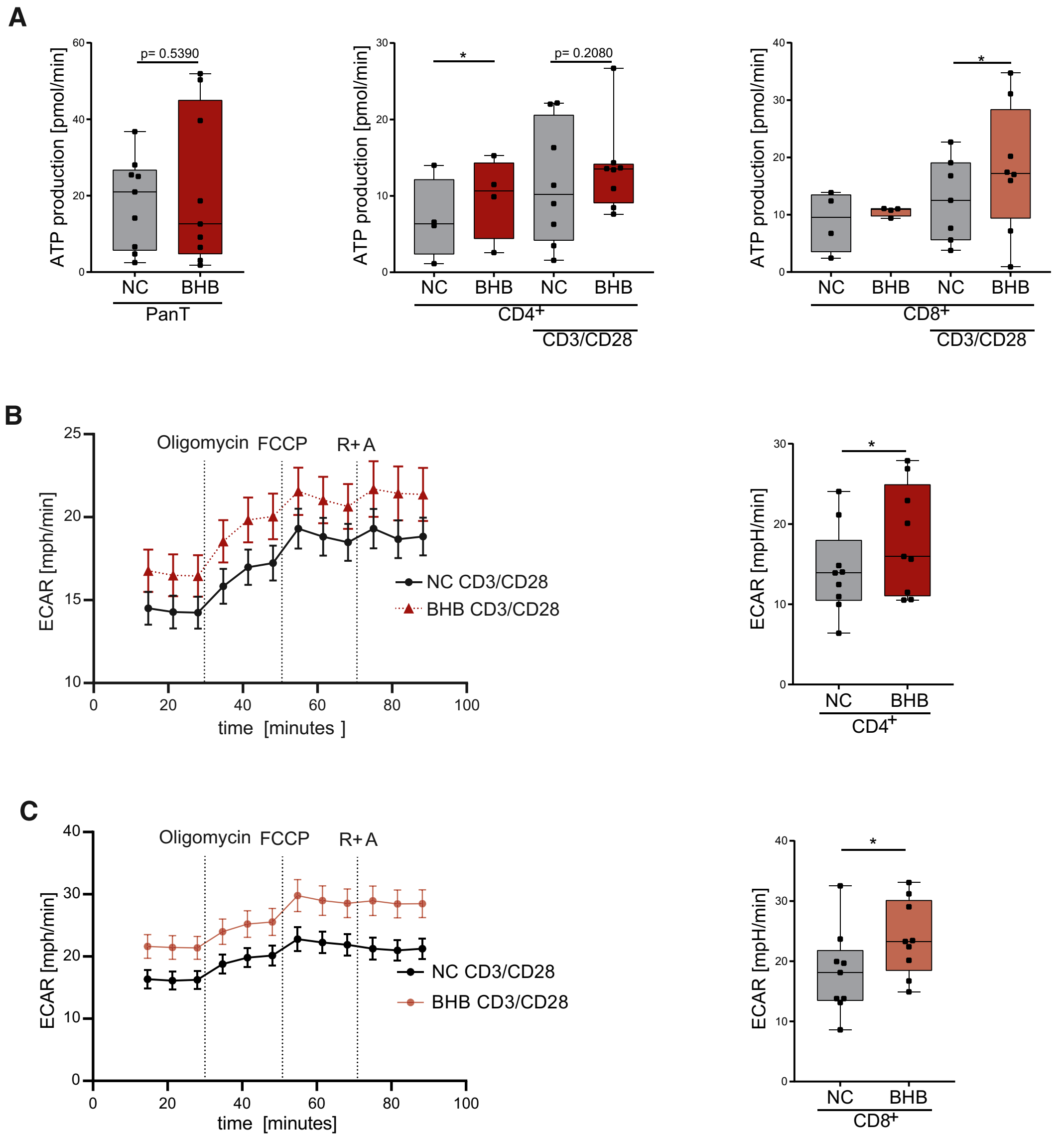

Figure EV1. Ketone bodies shift T-cell metabolism to oxidative phosphorylation.

Human PBMCs were cultivated for 48 h in RPMI containing 80 mg/dl glucose (NC) and supplemented with 10 mM beta-hydroxybutyrate (BHB). T-cell stimulation was performed through CD3/CD28 Dynabeads at a bead:cell ratio of 1:8. Pan T cells and CD4$^+$ and CD8$^+$ T cells were isolated through magnetic cell separation. Mitochondrial metabolism was analyzed for each subpopulation using a Seahorse XF96 Analyzer.

A Mitochondrial ATP production was measured in pan T cells, CD4$^+$ T cells, and CD8$^+$ T cells, n = 9 (pan T) and n = 4/8 (unstimulated/stimulated CD4 and CD8) individual experiments.

B, C Extracellular acidification rate, measured in (B) CD4$^+$ T cells and (C) CD8$^+$ T cells, n = 9 biological replicates.

Data information: Data depicted as mean ± SEM (ECAR) and box plots with median, 25th and 75th percentiles and range (all other). Dots indicating individual values. *P < 0.05 paired t-test/Wilcoxon matched-pairs signed rank test, as appropriate.

© 2021 The Authors

© 2021 The Authors

Figure EV2. BHB does not increase ROS in unstimulated human T cells.

Human peripheral blood mononuclear cells (PBMCs) were cultivated for 48 h in RPMI containing 80 mg/dl glucose (NC) and supplemented with 10 mM beta-hydroxybutyrate (BHB). Native T cells were analyzed via flow cytometry using the indicated dyes and antibodies identifying T cells and $CD4^+/CD8^+$ T cell subsets.

A Quantification of cellular ROS using CellROX, indicated by MFI FITC in human $CD4^+/CD8^+$ T cells, $n = 7/6$ biological replicates, $P = 0.4917/0.6324$.

B Mitochondrial superoxide production quantified through MitoSOX staining, depicted as MFI PE in human pan/$CD4^+/CD8^+$ T cells, $n = 6/11/12$ biological replicates, $P = 0.0938/0.3209/0.1401$.

C Representative gating strategy for memory cell quantification using flow cytometry.

D–F Representative histogram plots for quantification of (D) cellular ROS using CellROXgreen, (E) mitochondrial mass using MitoTracker green and (F) mitochondrial ROS using MitoSOXred.

G Densitometric analysis of Western blots of mitochondrial oxidative phosphorylation proteins in pan/$CD4^+/CD8^+$ T cells as indicated, $n = 3/4/4$ (CD4 complex IV, $n = 3$) biological replicates.

Data information: Data depicted as box plots with median, 25th and 75th percentiles and range, crosses indicating mean, dots indicating individual values. *$P < 0.05$, **$P < 0.01$, paired t-test/Wilcoxon matched-pairs signed rank test, as appropriate.

© 2021 The Authors

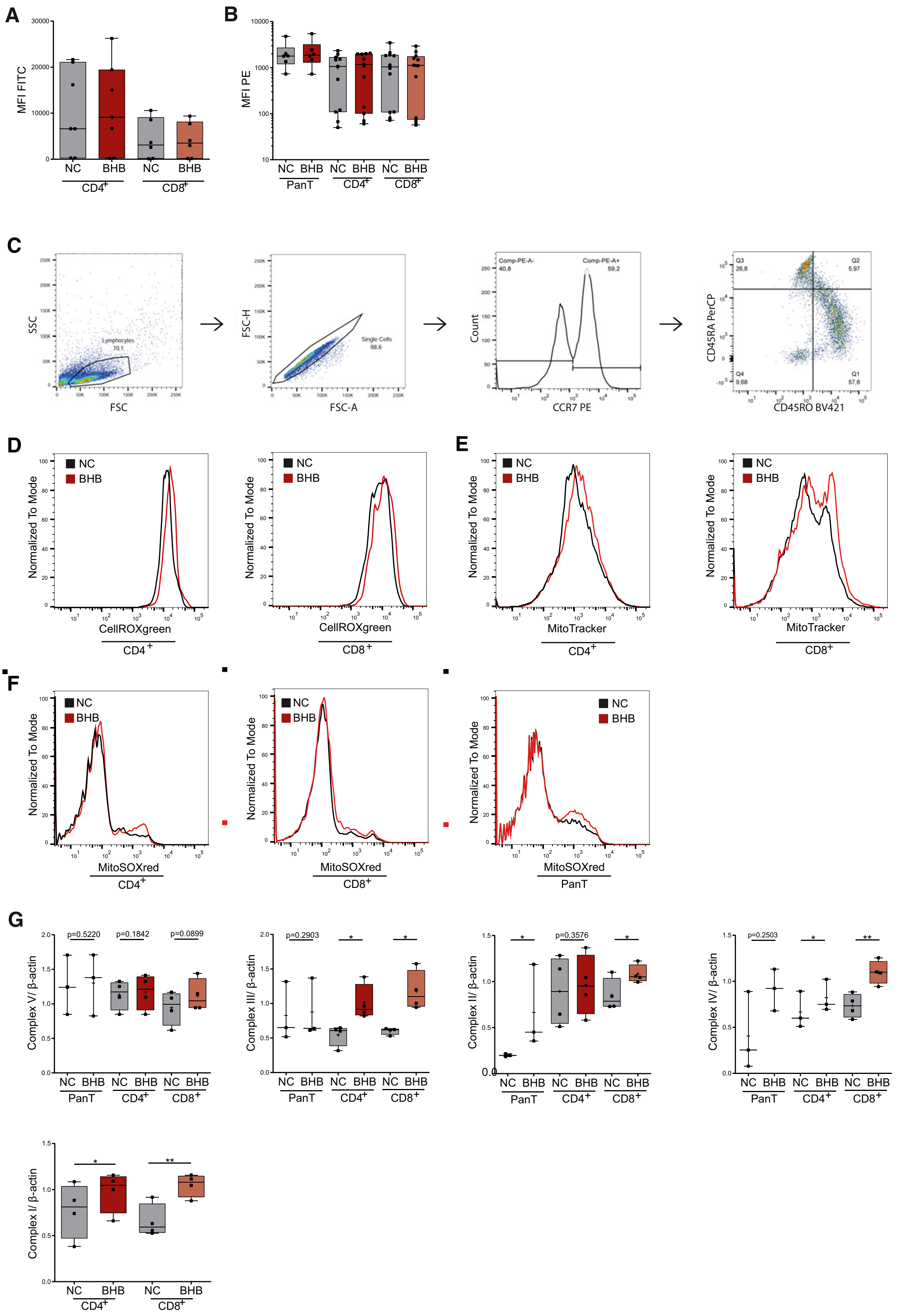

A
MFI FITC
NC BHB NC BHB
CD4+ CD8+
B
MFI PE
NC BHB NC BHB NC BHB
PanT CD4+ CD8+
C
SSC
FSC
Lymphocytes
70,1
FSC-H
FSC-A
Single Cells
98,6
Count
CCR7 PE
Comp-PE-A-
40,8
Comp-PE-A+
59,2
CD45RA PerCP
CD45RO BV421
Q3
26,8
Q2
5,97
Q4
9,68
Q1
57,6
D
Normalized To Mode
NC
BHB
CellROXgreen
CD4+
CD8+
E
MitoTracker
F
MitoSOXred
PanT
G
Complex V/ β-actin
Complex III/ β-actin
Complex II/ β-actin
Complex IV/ β-actin
Complex I/ β-actin
p=0.5220
p=0.1842
p=0.0899
p=0.2903
p=0.3576
p=0.2503

Figure EV2.

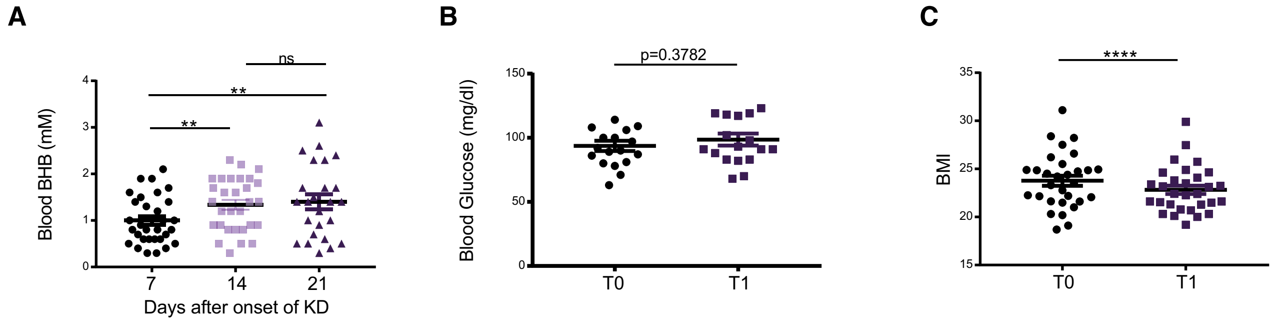

Figure EV3. Ketogenic diet *in vivo*.

Healthy volunteers conducted a 3-week ketogenic diet (KD) with limited carbohydrate consumption of < 30 g/day.

A Blood BHB concentration of healthy volunteers at the indicated time points as measured by point of care instruments.
B Blood glucose concentration of healthy volunteers was analyzed prior to starting the diet (T_0) and again after 3 weeks of strict adherence to the diet (T_1).
C Participants' BMI measured at the start and at the end of KD.

Data information: Data depicted as mean ± SEM, dots indicating individual values. $^{ns}P > 0.05$, $^{**}P < 0.01$, $^{****}P < 0.0001$, paired *t*-test/Wilcoxon matched-pairs signed rank test, as appropriate.

Figure EV4. Ketogenic diet primes human T cells to mitochondrial metabolism and memory cell development *in vivo*.

Forty-four healthy volunteers conducted a 3-week KD with a limited carbohydrate consumption of < 30 g/day. Blood was taken and analyzed prior to starting the diet (T_0) and again after 3 weeks of strict adherence to the diet (T_1). PBMCs were isolated. If applicable, T-cell stimulation was performed through CD3/CD28 Dynabeads at a bead:cell ratio of 1:8. Pan/$CD4^+$/$CD8^+$ T cells were separated via magnetic cell labeling. Mitochondrial metabolism was analyzed for each subpopulation using a Seahorse XF96 Analyzer.

A Mitochondrial ATP production was measured in stimulated $CD4^+$ and $CD8^+$ T cells, $n = 14$ individual human subjects.
B–E Extracellular acidification rate, measured in (B) unstimulated and (C) stimulated $CD4^+$ T cells and (D) unstimulated and (E) stimulated $CD8^+$ T cells, $n = 6$ individual human subjects.
F, G Glycolytic proton efflux rate (glycoPER) and compensatory (maximum) glycoPER measured in stimulated (F) $CD4^+$ T cells and (G) $CD8^+$ T cells, depicted as mean ± 95% CI/mean ± SEM, $n = 7/8$ individual human subjects.
H Quantification of mitochondrial membrane potential using JC1, indicated by MFI PE/FITC in human lymphocytes *in vivo*. $n = 4/5$ (unstimulated/stimulated) individual human subjects.
I, J Quantification of (I) cellular and (J) mitochondrial ROS using CellROX/MitoSOX, indicated by MFI FITC and MFI PE in native human Pan/$CD4^+$/$CD8^+$ T cells, $n = 8$ (CellROX), 10/9/10 (MitoSOX) individual human subjects.
K Quantification of mitochondrial mass using MitoTracker green, indicated by MFI FITC in native human pan/$CD4^+$/$CD8^+$ T cells, $n = 10/10/11$ individual human subjects.
L TruCulture Interleukin (IL)1α/β protein quantification of LPS-stimulated whole blood samples, $n = 11/12$ individual human subjects.
M TruCulture IL8 and IL12 subunit p40 protein quantification of unstimulated whole blood samples, $n = 5$ (IL8: T0 $n = 4$) individual human subjects. TruCulture IFNγ, IL4, IL6, IL23, and TNFα protein quantification of unstimulated whole blood samples were below the lower limit of quantification.

Data information: Data depicted as mean ± SEM (ECAR/glycoPER) and box plots (all other) with median, 25th and 75th percentiles and range, dots indicating individual values. $^{*}P < 0.05$, $^{**}P < 0.01$, paired *t*-test/Wilcoxon matched-pairs signed rank test, as appropriate.

 © 2021 The Authors

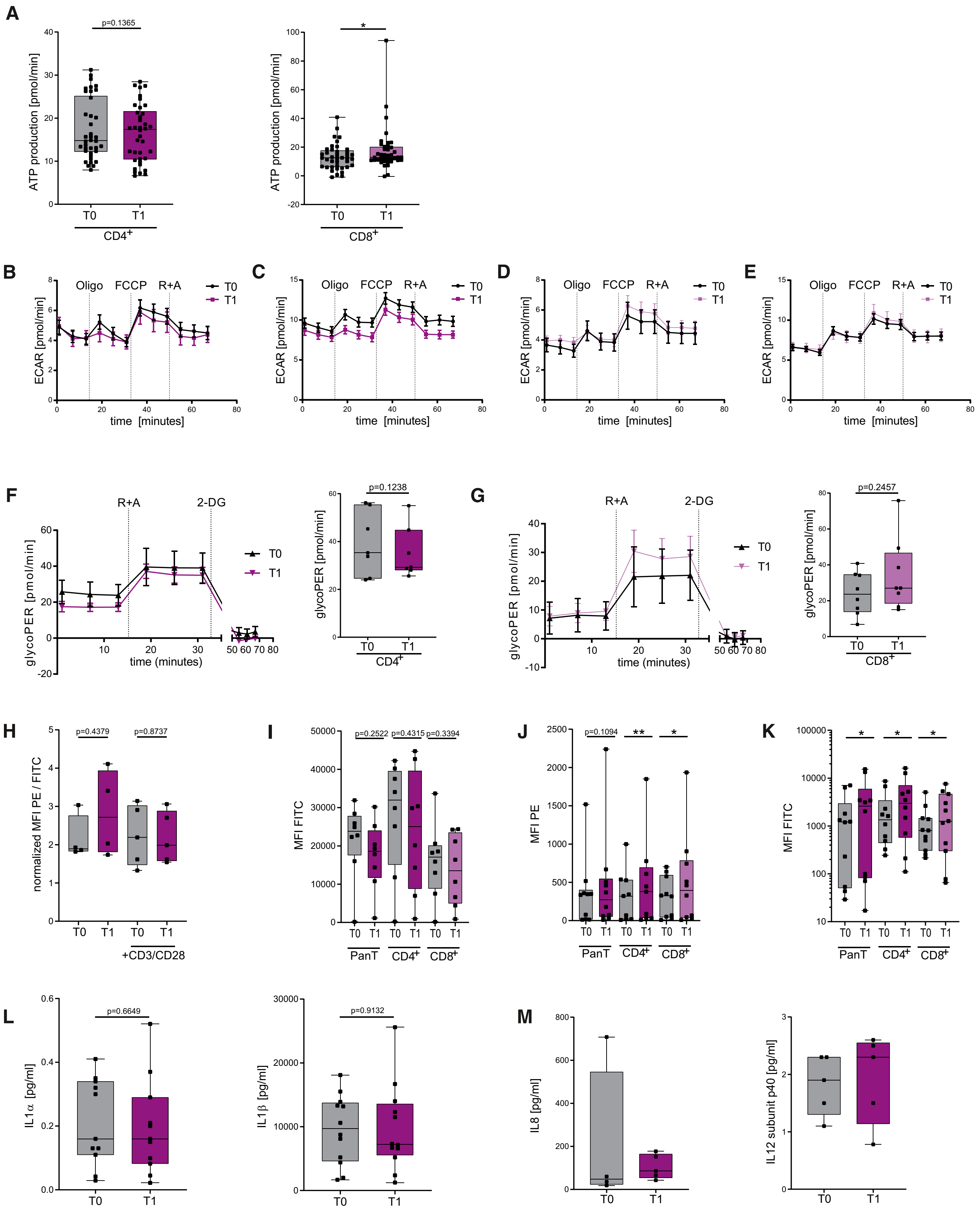
A
p=0.1365
ATP production [pmol/min]
T0
T1
CD4+
*
ATP production [pmol/min]
T0
T1
CD8+
B
Oligo FCCP R+A
T0
T1
ECAR [pmol/min]
time [minutes]
C
Oligo FCCP R+A
T0
T1
ECAR [pmol/min]
time [minutes]
D
Oligo FCCP R+A
T0
T1
ECAR [pmol/min]
time [minutes]
E
Oligo FCCP R+A
T0
T1
ECAR [pmol/min]
time [minutes]
F
R+A
2-DG
T0
T1
glycoPER [pmol/min]
time (minutes)
p=0.1238
glycoPER [pmol/min]
T0
T1
CD4+
G
R+A
2-DG
T0
T1
glycoPER [pmol/min]
time (minutes)
p=0.2457
glycoPER [pmol/min]
T0
T1
CD8+
H
p=0.4379
p=0.8737
normalized MFI PE / FITC
T0
T1
T0
T1
+CD3/CD28
I
p=0.2522
p=0.4315
p=0.3394
MFI FITC
T0 T1 T0 T1 T0 T1
PanT CD4+ CD8+
J
p=0.1094
**
*
MFI PE
T0 T1 T0 T1 T0 T1
PanT CD4+ CD8+
K
*
*
*
MFI FITC
T0 T1 T0 T1 T0 T1
PanT CD4+ CD8+
L
p=0.6649
IL1α [pg/ml]
T0
T1
p=0.9132
IL1β [pg/ml]
T0
T1
M
IL8 [pg/ml]
T0
T1
IL12 subunit p40 [pg/ml]
T0
T1

Figure EV4.
