# Supplementary material for: Very‐low‐carbohydrate diet enhances human T‐cell immunity through immunometabolic reprogramming
Source: EMBO Mol Med. 2021 Jun 21;13(8):e14323. doi: 10.15252/emmm.202114323 (PMC8350890; doi:10.15252/emmm.202114323)

Source Data – FIGURE 3

Figure 3c unprocessed images

CD4 NC

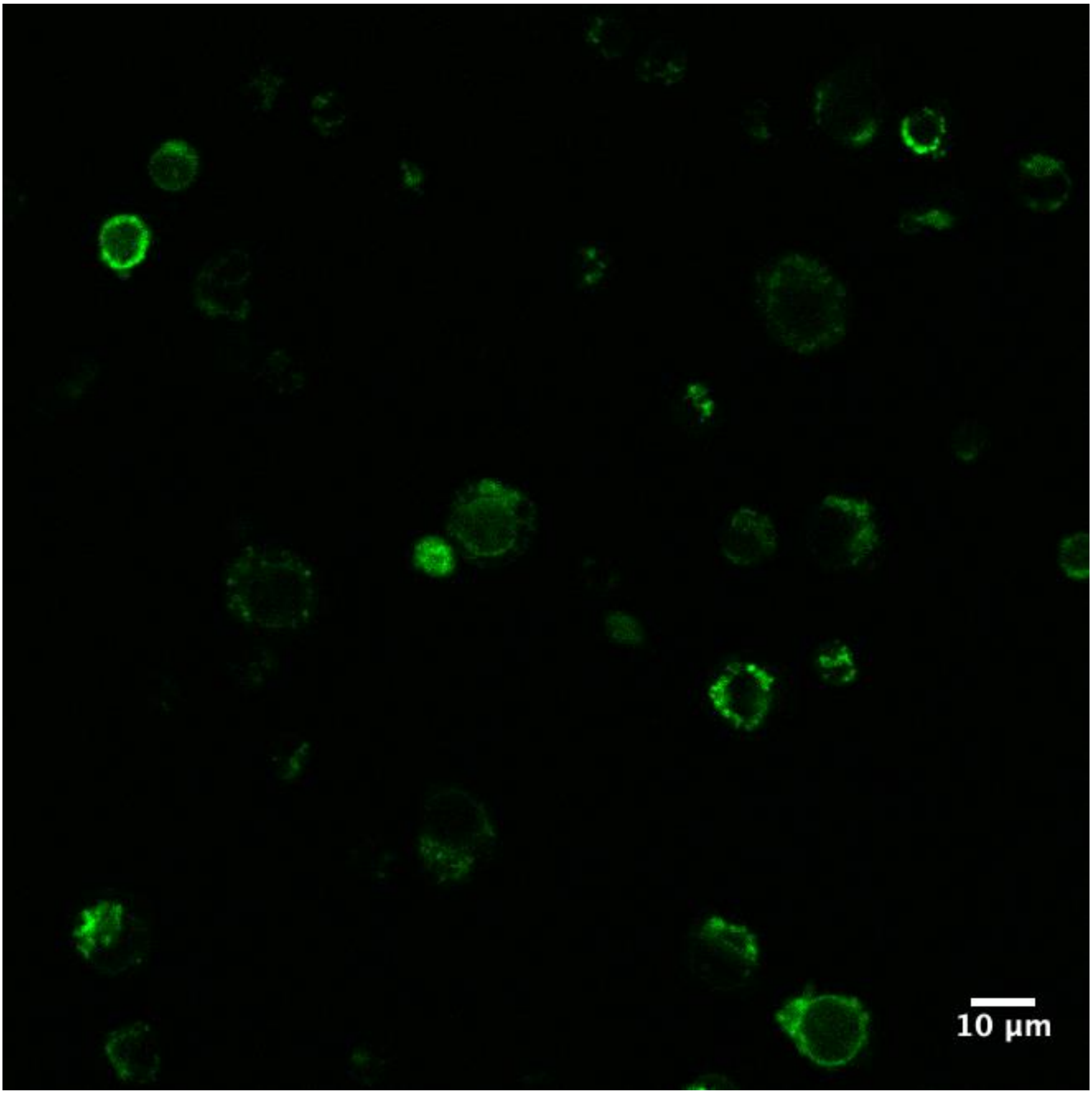

CD4 BHB

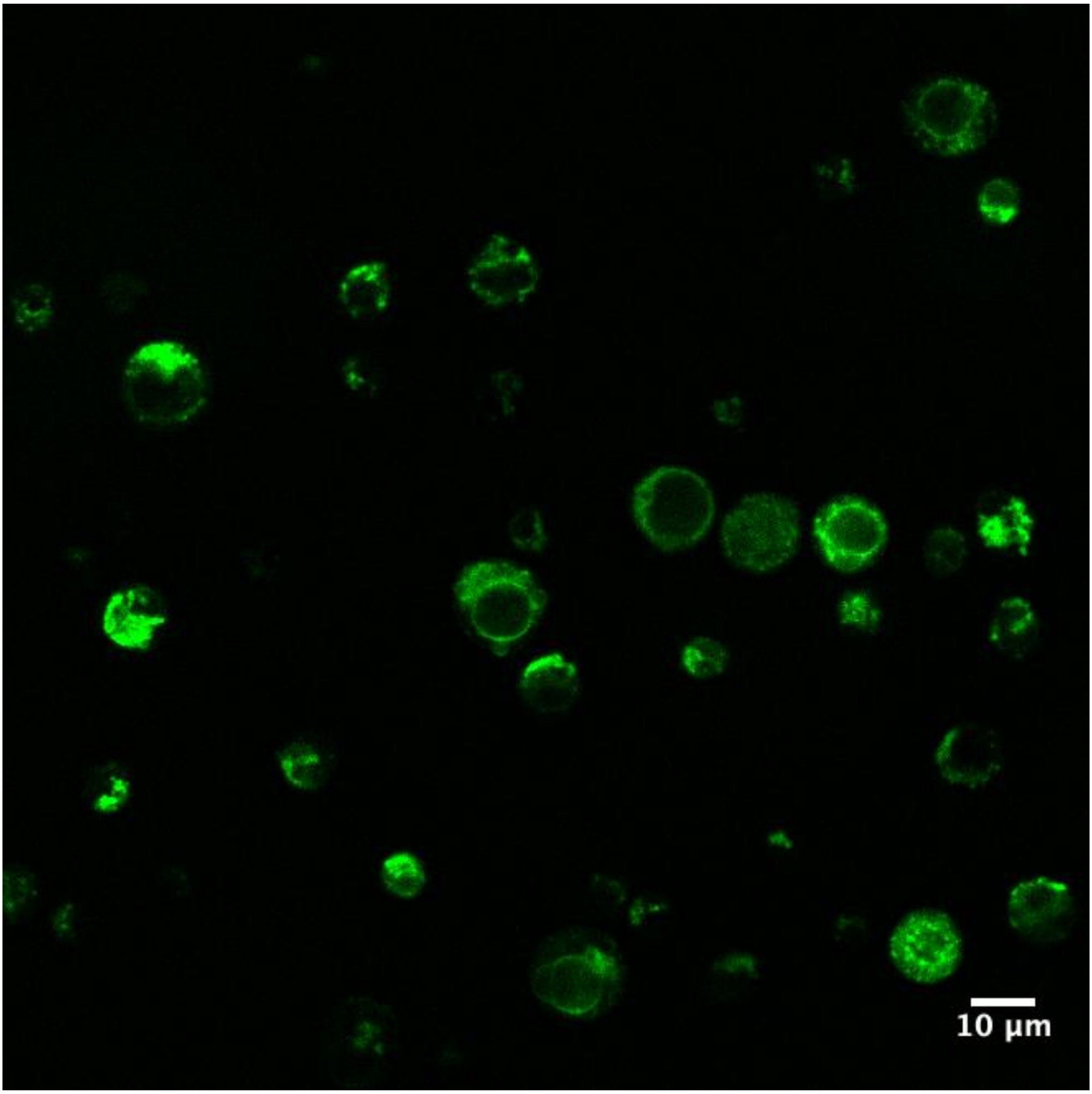

CD8 NC

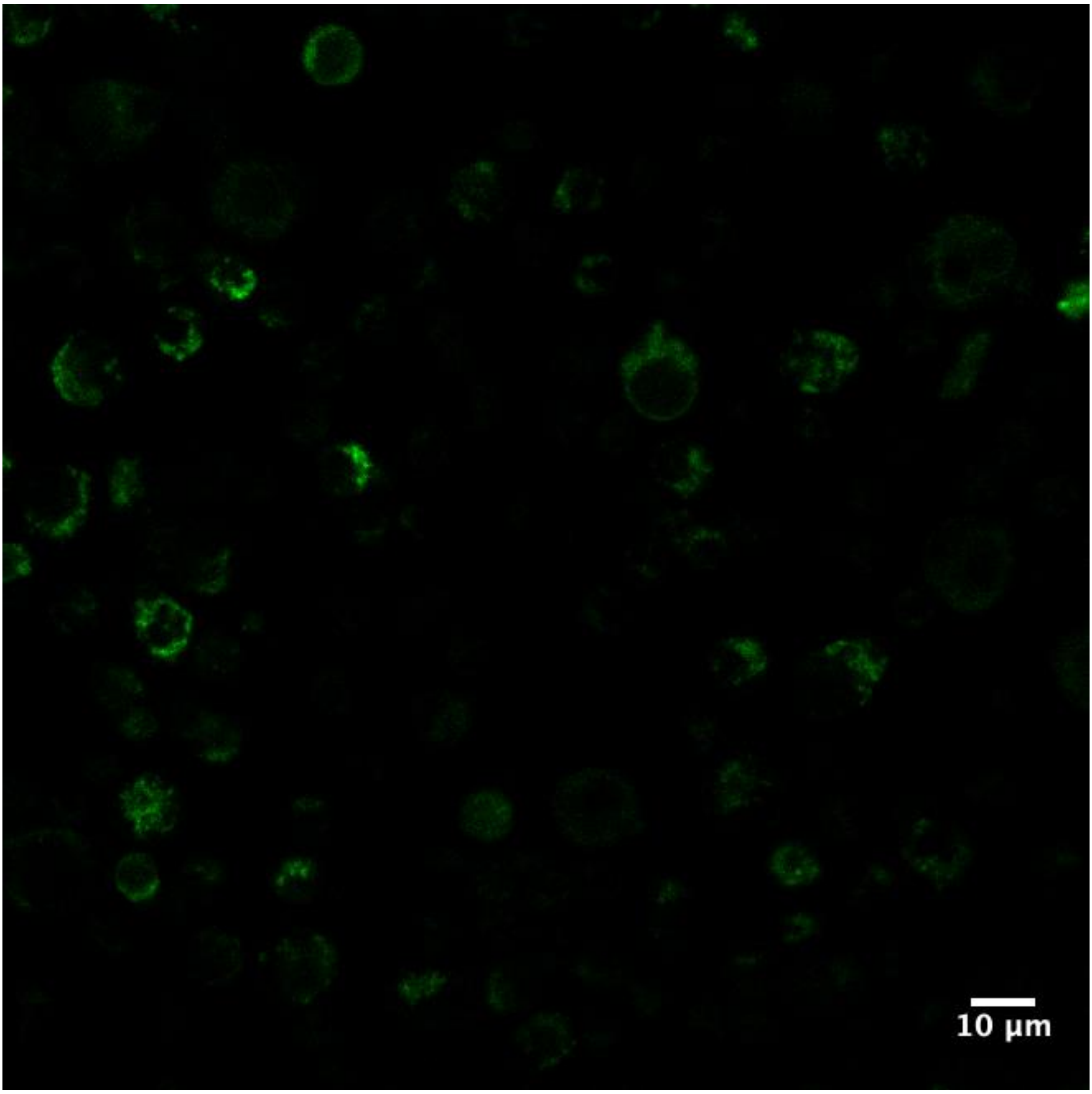

CD8 BHB

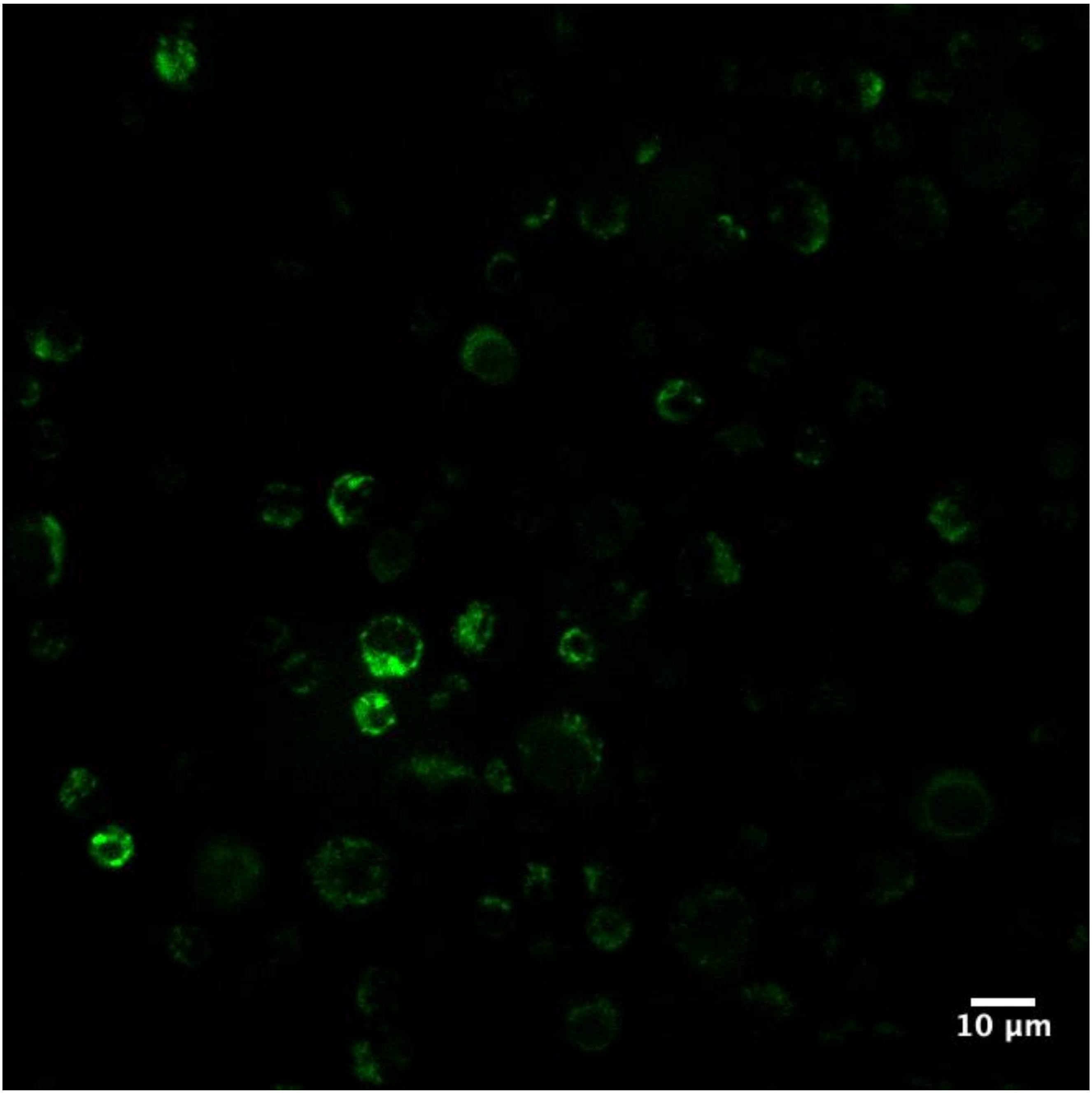

Figure 3e Pan T (left panel)

Overview and molecular weight marker

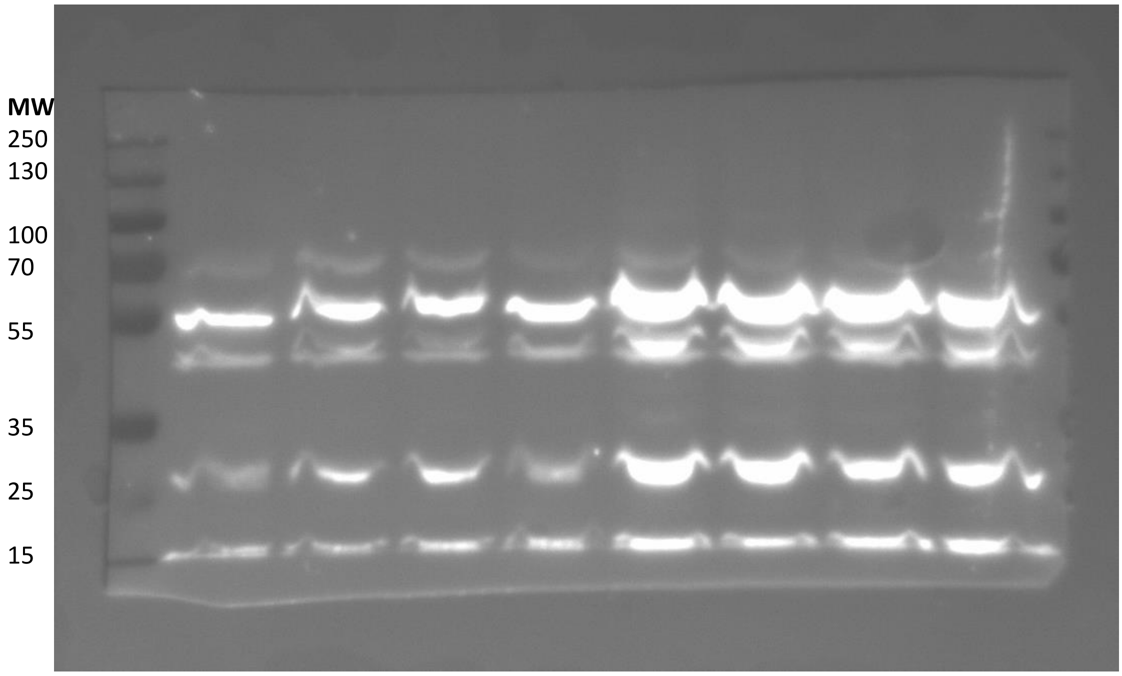

Uncropped and unprocessed scans (OXPHOS + bActin)

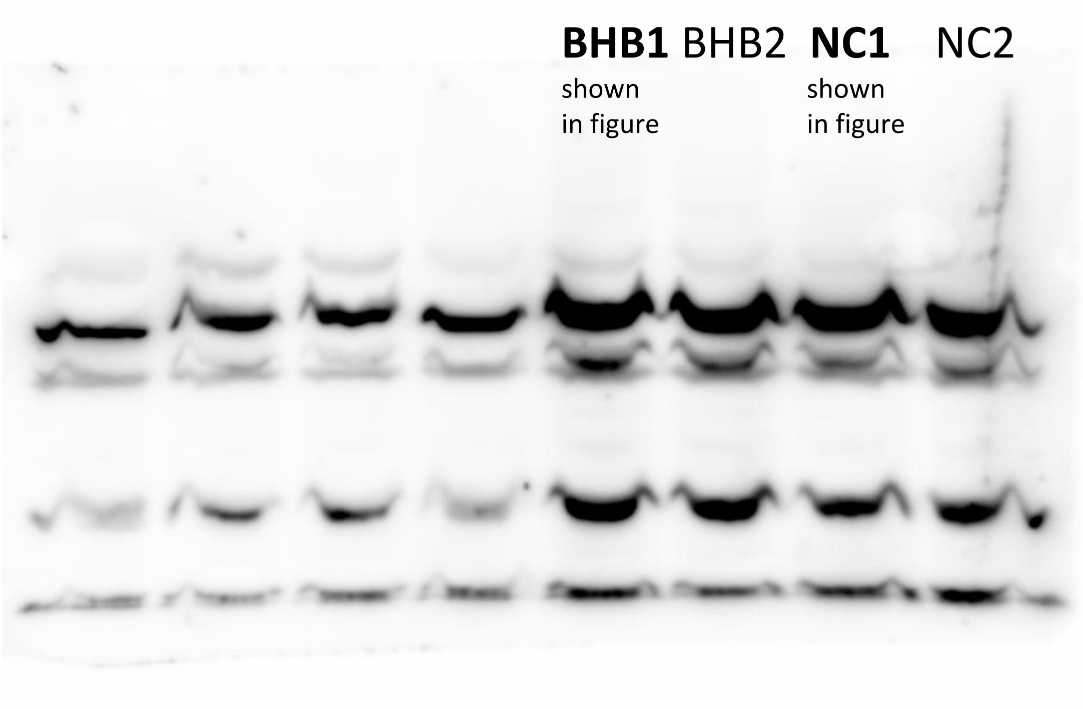

Figure 3e Pan T (left panel)

Uncropped and unprocessed scans (bActin)

BHB1 BHB2 **NC1** NC2

shown in figure (BHB1); shown in figure (NC1)

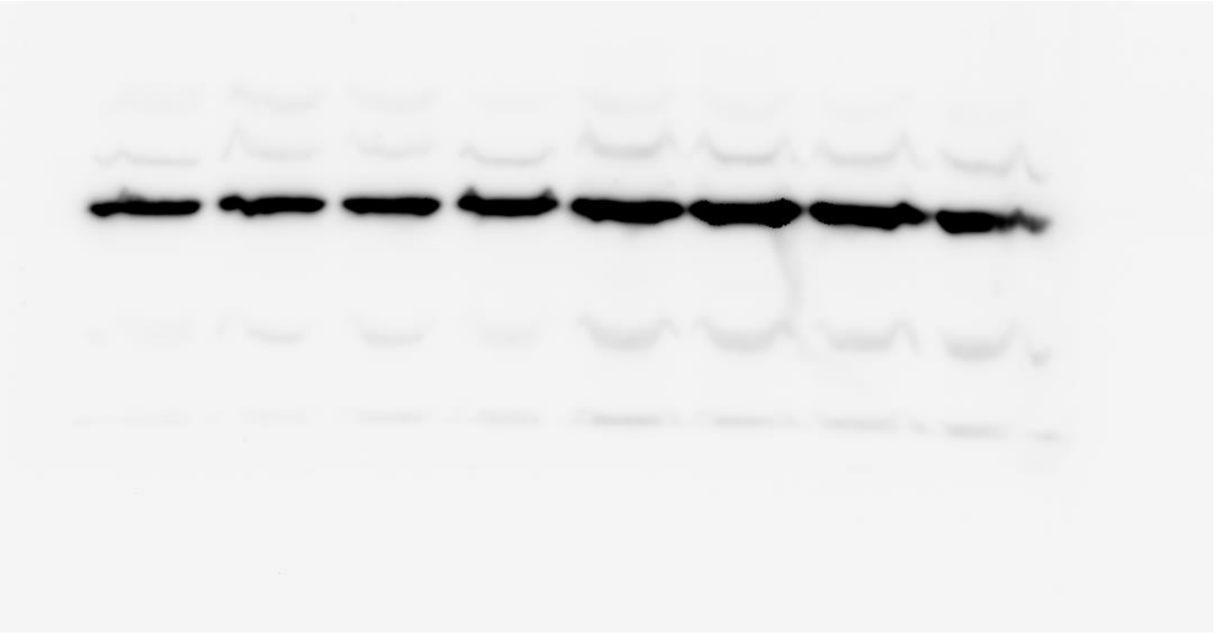

Figure 3e CD4 (mid panel)

Overview and molecular weight marker

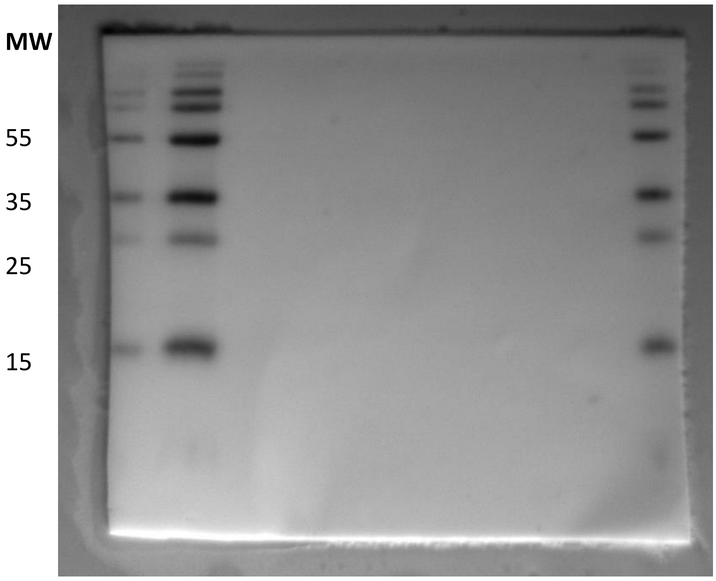

Uncropped and unprocessed scans (OXPHOS + bActin)

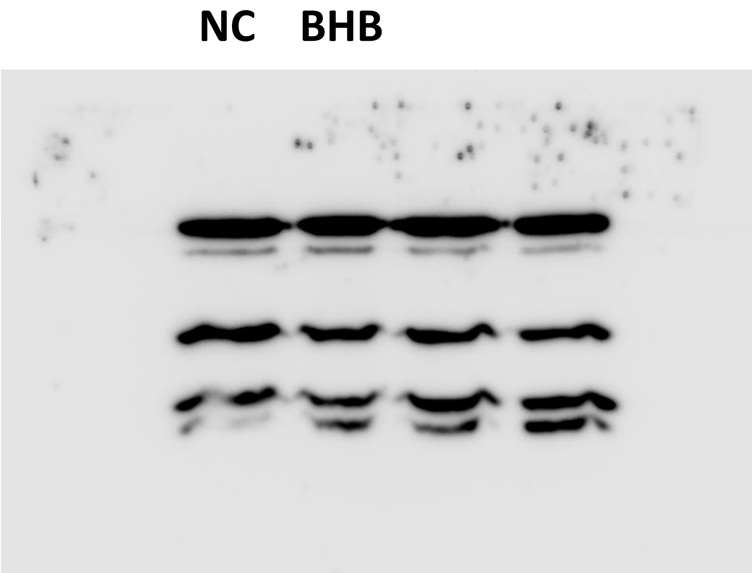

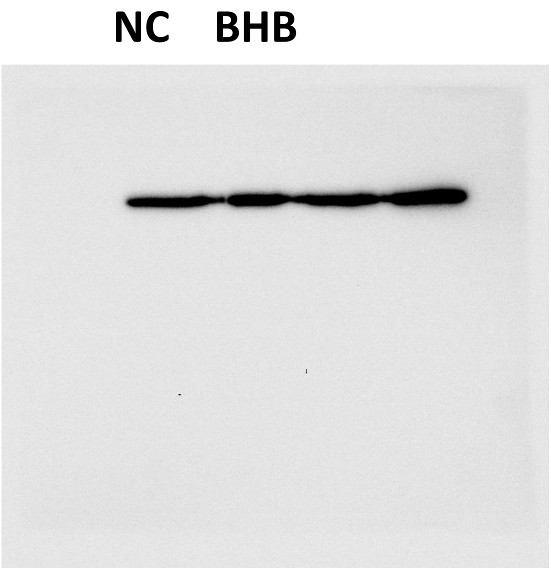

Figure 3e CD8 (right panel)

Overview and molecular weight marker

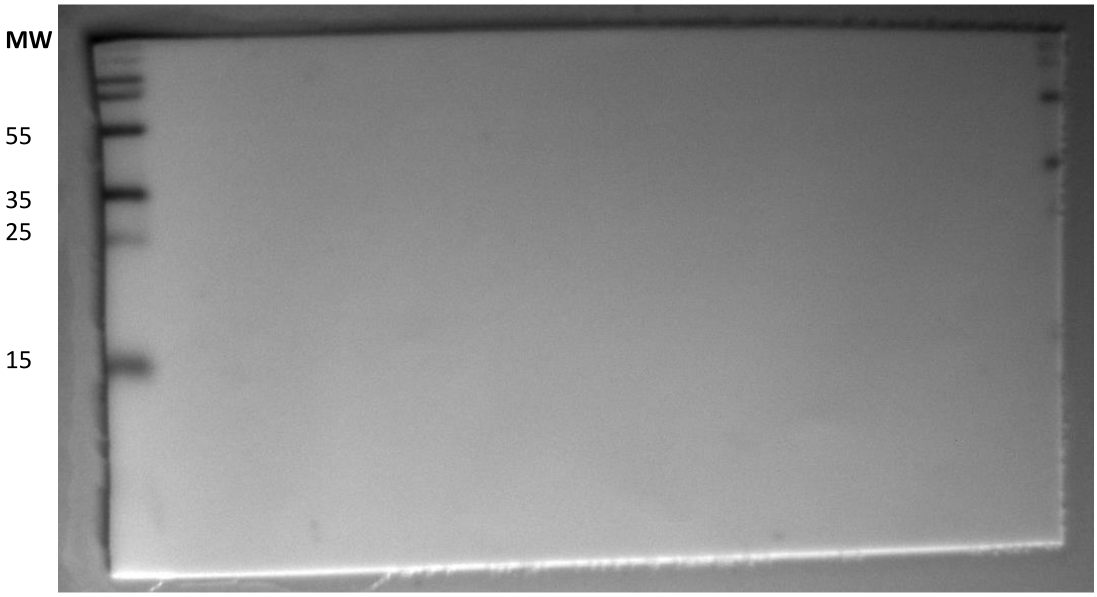

Uncropped and unprocessed scans (OXPHOS + bActin)

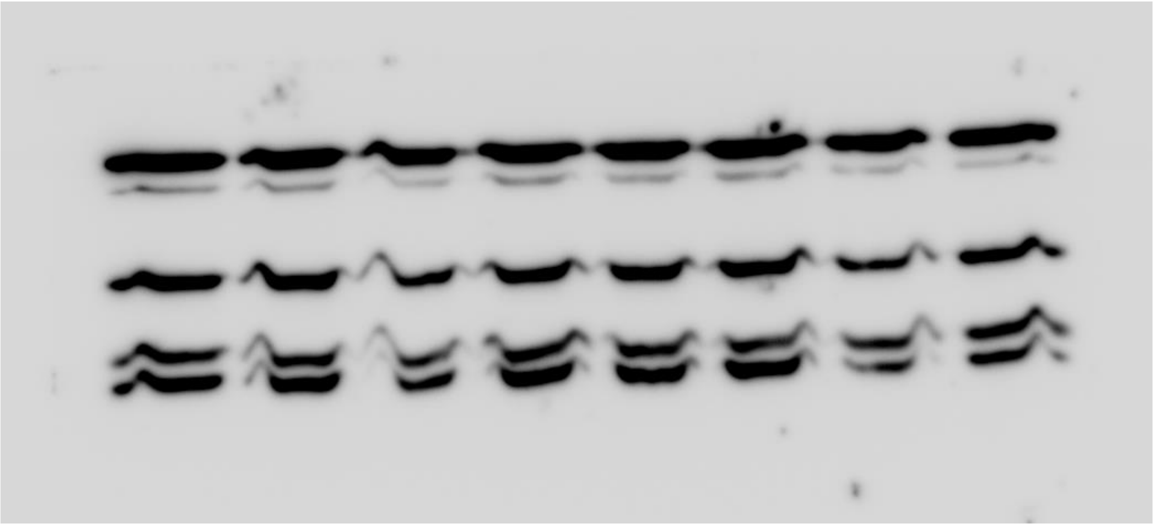

NC BHB

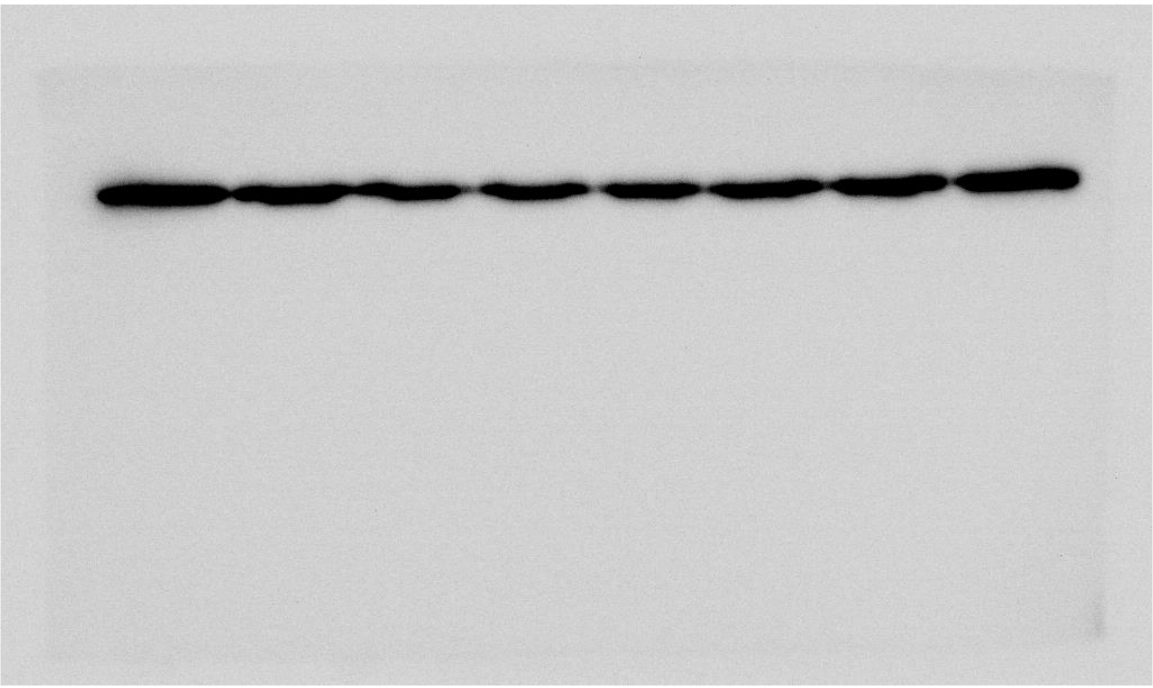

Supplement: Supplementary file 4 — Source Data for Figure 3 [file EMMM-13-e14323-s004.pdf]
