# Supplementary material for: Very‐low‐carbohydrate diet enhances human T‐cell immunity through immunometabolic reprogramming
Source: EMBO Mol Med. 2021 Jun 21;13(8):e14323. doi: 10.15252/emmm.202114323 (PMC8350890; doi:10.15252/emmm.202114323)

Source Data – FIGURE 6

Figure 6e

Overview and molecular weight marker

MW
55
35
25
15

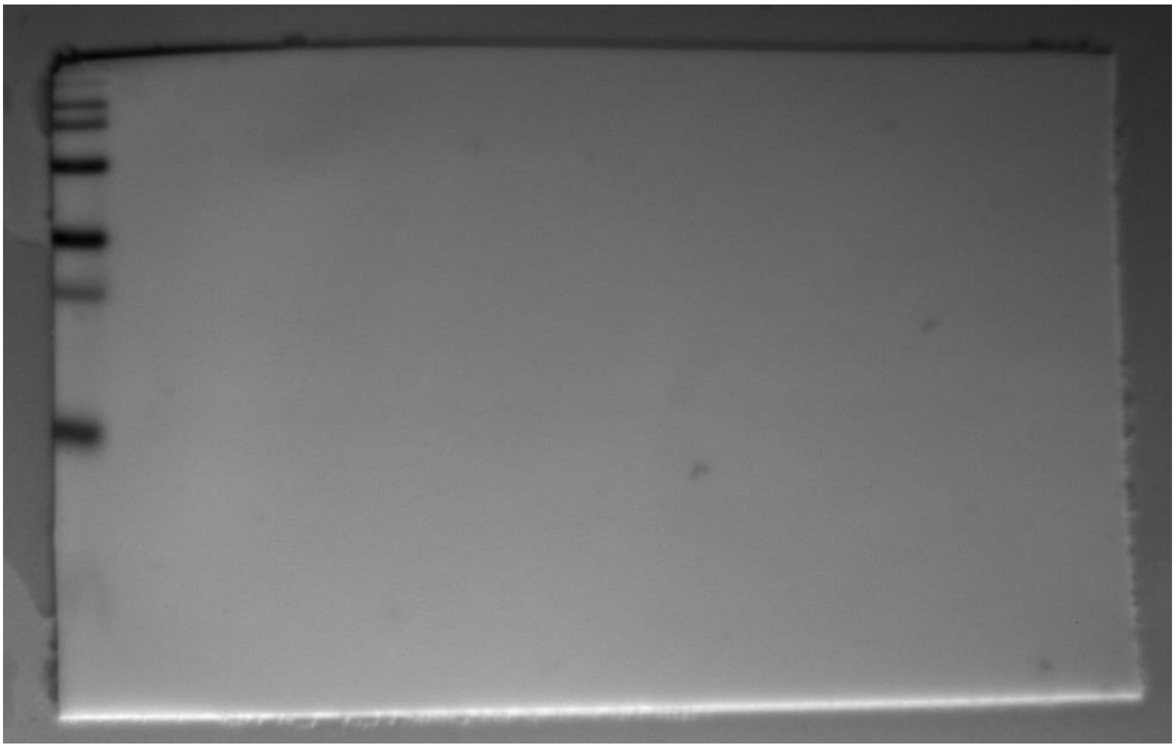

Uncropped and unprocessed scans (OXPHOS)

CD4 | CD8
T0 T1 | T0 T1

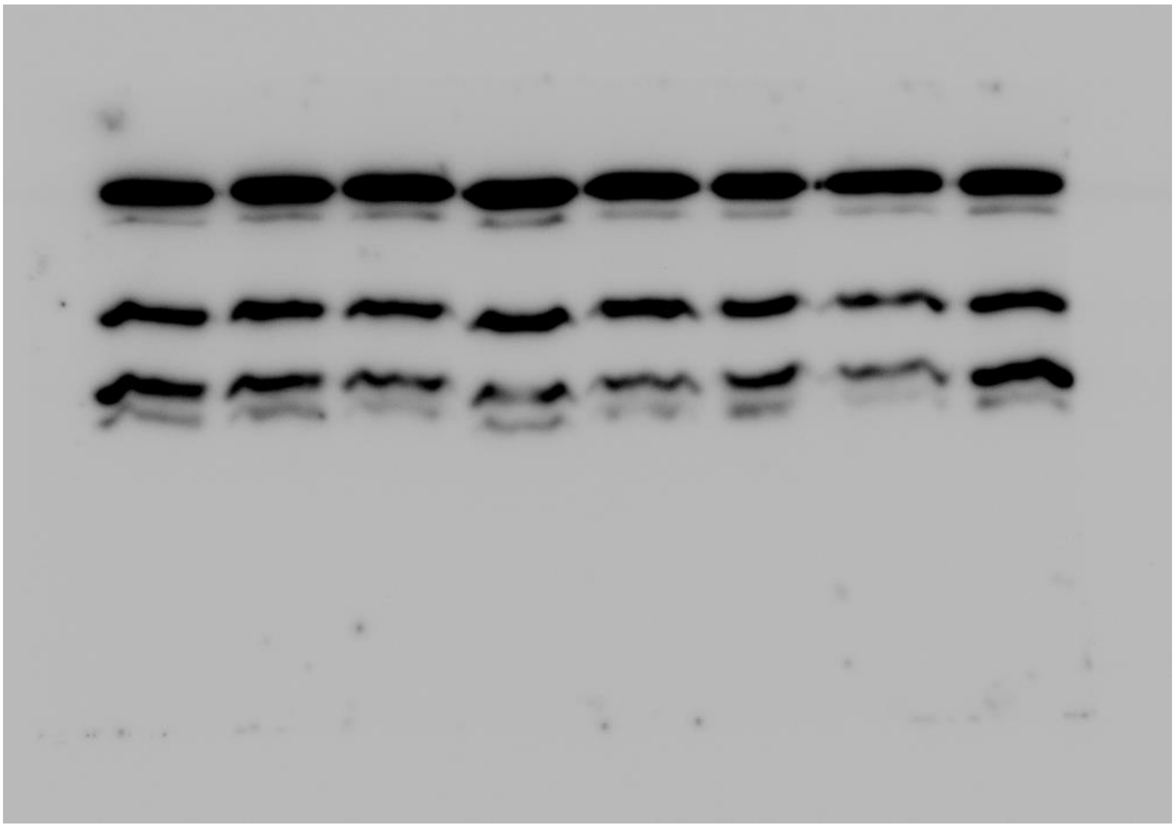

Figure 6e

Uncropped and unprocessed scans (bActin)

CD4 T0 T1 CD8 T0 T1

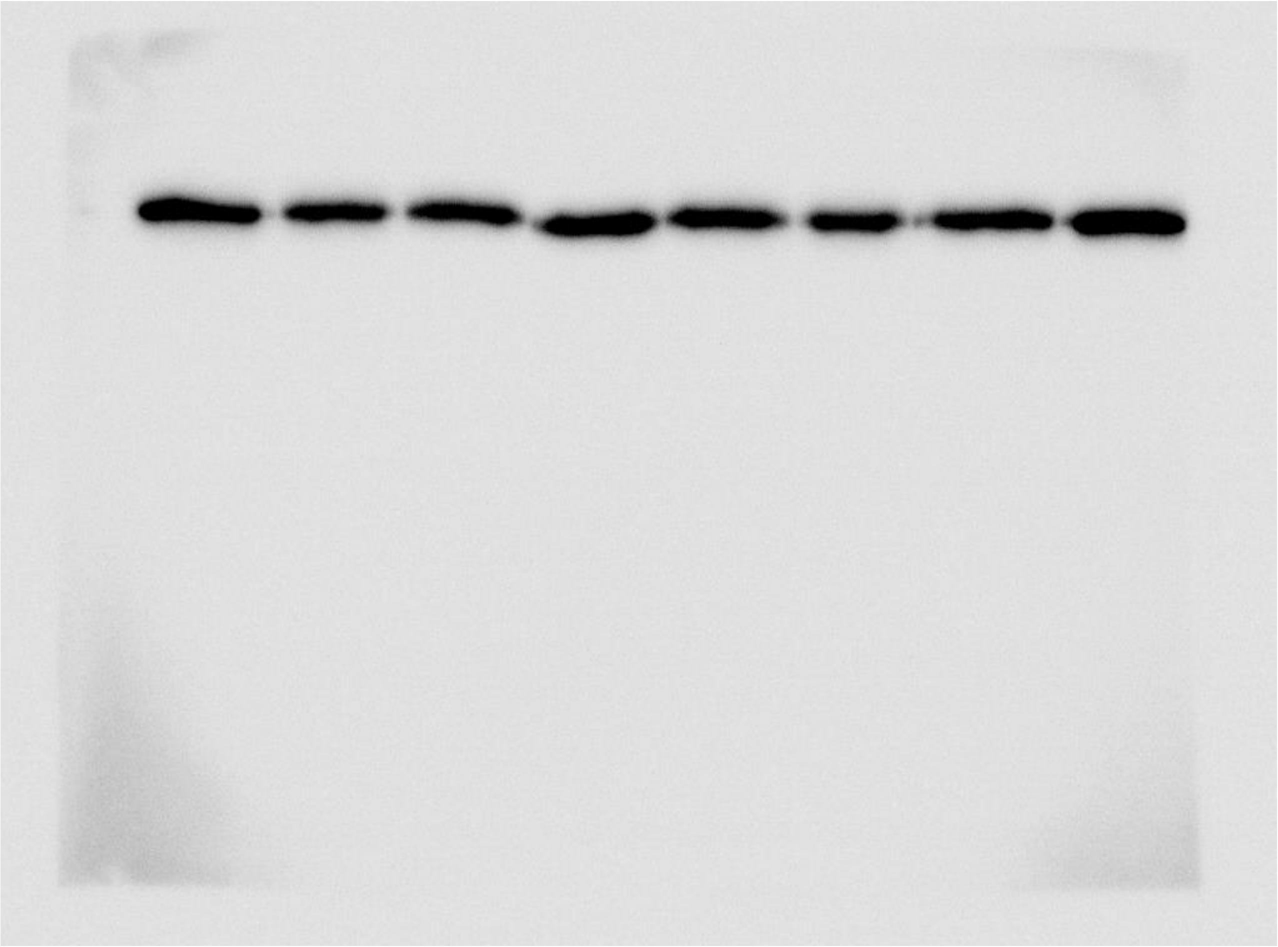

Figure 6f unprocessed images

CD4 T0

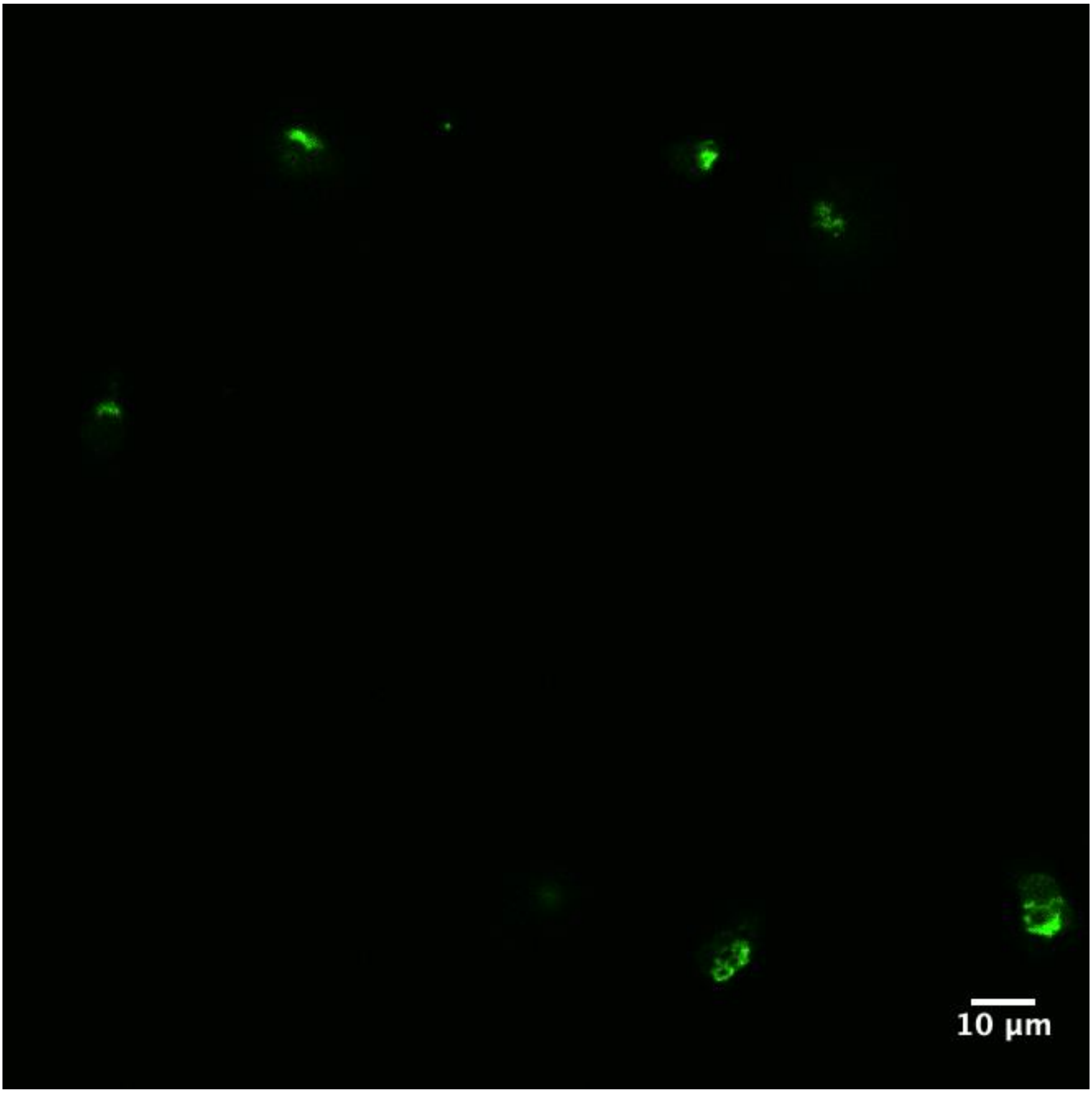

CD4 T1

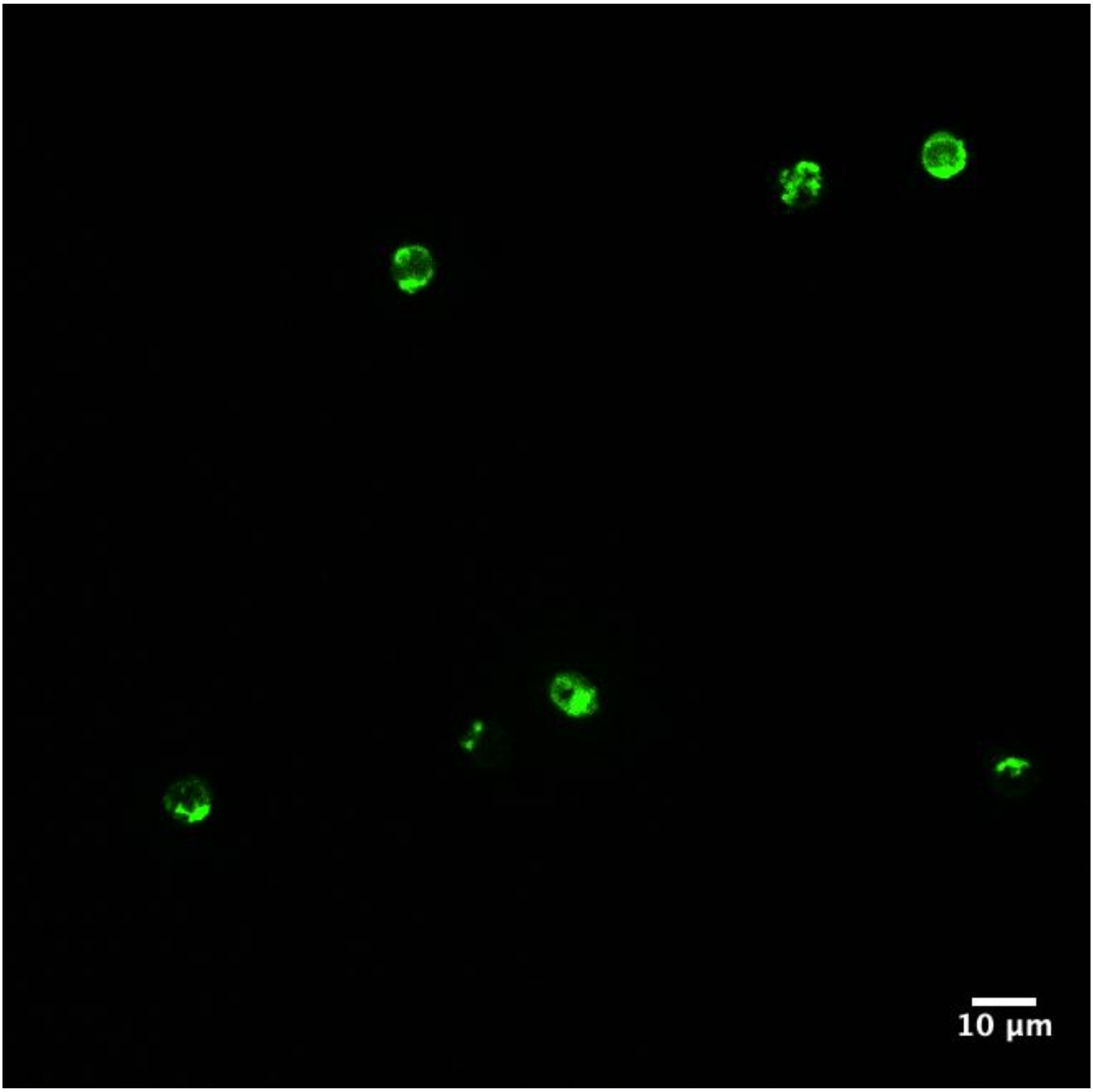

CD8 T0

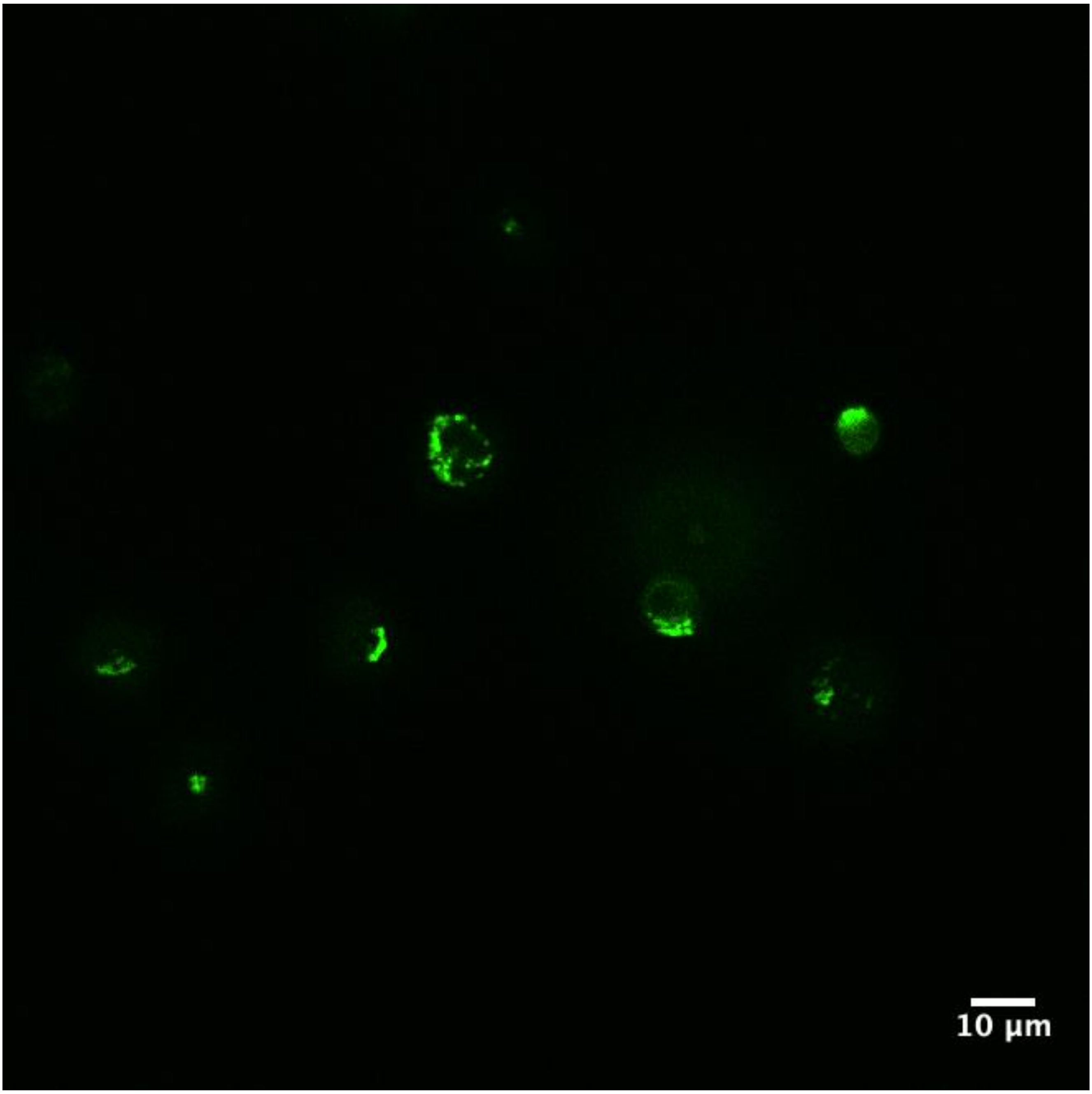

CD8 T1

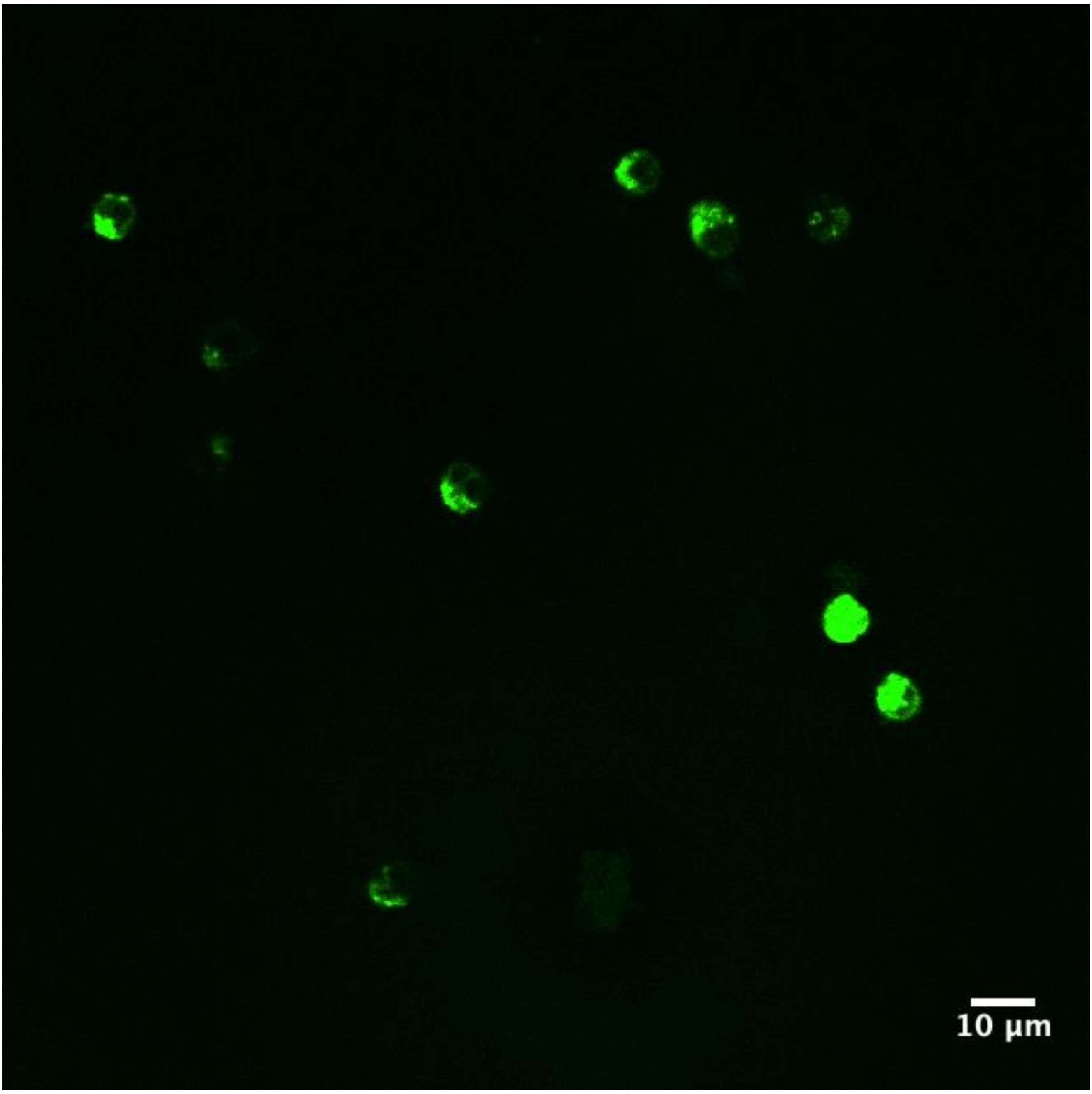

Supplement: Supplementary file 5 — Source Data for Figure 6 [file EMMM-13-e14323-s002.pdf]
